# Urine PD-L1 as a non-invasive biomarker for immune checkpoint inhibitor (ICI) therapy in bladder cancer

**DOI:** 10.1016/j.abst.2025.05.001

**Published:** 2025-05-31

**Authors:** Qianyun Ge, Peng Wang, Shang-jui Wang, Akshay Sood, Lingbin Meng, Cheryl Lee, Anil V. Parwani, Jenny Li, Xuefeng Liu

**Affiliations:** a Comprehensive Cancer Center, Ohio State University, Columbus, OH, USA; b Department of Medicine, Wexner Medical Center, Ohio State University, Columbus, OH, USA; c Department of Radiation Oncology, Wexner Medical Center, Ohio State University, Columbus, OH, USA; d Department of Urology, Wexner Medical Center, Ohio State University, Columbus, OH, USA; e Departments of Pathology, College of Medicine, The Ohio State University, Columbus, OH, USA; f Departments of Pathology, Urology and Radiation Oncology, Wexner Medical Center, Ohio State University, Columbus, OH, USA

**Keywords:** uPD-L1, Bladder cancer, Non-invasive detection, Diagnostic tool, Prognostic tool

## Abstract

Bladder cancer (BCa) is a common urological malignancy with a high recurrence rate, often within 2 years of initial diagnosis and treatment. Due to this high recurrence, near all patients require cystoscopic surveillance, which is invasive, uncomfortable, and costly. The cost of surveillance makes this cancer the most expensive cancer per case among all cancer types in the US. Therefore, early detection of recurrence or assessment of patients’ response to treatment, particularly through non-invasive methods, is urgently needed. Since immune checkpoint inhibitors (ICIs) are widely used in many clinical trials for BCa treatment, having non-invasive and reliable biomarkers to select appropriate patients for ICI therapies or predict their treatment responses would be invaluable. Here we summarized the potential applications of programmed death-ligand 1 (PD-L1) from urine or urine BCa cell samples in BCa clinical settings. We discuss the use of both the free form of PD-L1 in urine samples and the expression levels of PD-L1 on the BCa cells shed in urine samples. Free PD-L1 can be measured with flow cytometry or ELISA-based approaches, while detecting PD-L1 on BCa cell surface requires isolating the urine-derived cancer cells and analyzing them via flow cytometry. Furthermore, we discuss the promising future research areas of urinary PD-L1 (uPD-L1) in bladder cancer, with a particular focus on the combination of conditional reprogramming cells (CRCs) technology and uPD-L1 studies, followed by an overview of several ongoing research topics. Based on current findings, uPD-L1 shows great potential as a versatile biomarker; however, further research is urgently needed to facilitate its translation into clinical applications.

## 1. Introduction

Bladder cancer (BCa) is among the top four most common cancers in men in the United States, leading to approximately 81,400 new cases (4th) and 17,980 deaths (8th) in 2024.^1^ At diagnosis, approximately 75 % of BCa cases are diagnosed as the non-muscle-invasive BCa (NMIBC) stages, that are usually treated with transurethral resection (TUR) followed by intravesical instillation of therapeutic agents in high-risk patients. Despite this approach, the cancer recurrence rate remains high—approximately 50–60 % within two years—with disease progression to more advanced stages occurring in some patients. Due to the high recurrence rate, nearly all patients require cystoscopic surveillance, which is invasive, uncomfortable, and costly. The cost of ongoing surveillance makes bladder cancer the most expensive cancer to manage on a per-case basis among all cancer types in the United States. For those patients diagnosed with muscle-invasive BCa (MIBC), neoadjuvant chemotherapy induces a complete response in 30–40 % of cases, with these patients achieving a 5-year overall survival (OS) rate of approximately 85 %. In contrast, patients with residual invasive disease after neoadjuvant therapy have a significantly lower overall OS of only 35 %. For metastatic BCa, the new standard systemic therapy, EV-P, combines a Nectin-4 directed antibody-drug conjugate (enfortumab vedotin) and a programmed cell death-1 (PD-1) inhibitor (pembrolizumab), has shown promising results, with a median OS of 31.5 months. This is notably higher than the 16.1-month median OS observed with traditional cisplatin-based chemotherapy. Single ICI targeting either programmed cell death-1 (PD1) or programmed cell death ligand 1 (anti-PD-L1) immune checkpoint^2–4^ have demonstrated a response rate of approximately 20 %.^3,5,6^ Targeted therapy with erdafitinib, an inhibitor of fibroblast growth factor receptor 3 (FGFR3), has shown a response rate of around 40 % in BCa patients, with a median OS of 13.8 months.^7^ Although the therapeutic landscape in BCa is becoming optimistic, nearly all patients will eventually develop resistance. To date, there remains a lack of reliable tools to predict therapeutic response and to advance precision therapy in this disease. Higher PD-L1 expression on the tumor cell surface, as assessed with immunohistochemical (IHC), is associated with an increased response rate to ICI therapy,^3,8–10^ however the PD-L1 expression levels lack sufficient sensitivity and specificity to reliably predict response to ICI therapy. Although ctDNA shows promise in identifying patients who may benefit from adjuvant Atezolizumab therapy following radical cystectomy, its clinical utility remains under investigation in the IMVIGOR 11 trial.^11,12^ Currently, there is no reliable test to predict response to systemic therapy. Therefore, prognostic and predictive models with high discriminative accuracy are urgently needed to facilitate the individualized decision-making and improve clinical outcomes for BCa patients.

## 2. Immunotherapy of BCa

Of intravesical instillations, *Mycobacterium bovis* Bacillus Calmette-Guerin (BCG) is the most commonly used immunotherapy for BCa. It functions by activating both innate and adaptive immune responses and remains the first-line therapy for NMIBC group.^13,14^ BCG treatment consists of 6 weekly instillations followed by maintenance instillations. It demonstrates a high initial complete response rate in patients, with a 32 % reduction of cancer recurrence compared to intravesical chemotherapy instillations.^15,16^ However, BCG-treatment does not completely prevent disease progression, as evidenced by post-BCG recurrence. In fact, 30 %–40 % of patients experience BCG-treatment failure, particularly in cases with higher disease stages.^16,17^ These failures may be attributed to tumor immunosuppressive microenvironment.^16,18,19^

ICIs, which block the interaction between ligands and their matched receptors to restore the immune response against tumor cells, have emerged as a promising approach for BCa, this is especially true for the monoclonal antibodies against PD-1/PD-L1.^20^ Moreover, since BCG treatment has been proven to upregulate PD-L1 expression, ICIs are considered to have the potential to enhance BCG treatment or manage post-BCG recurrence.^21^ Currently, ICIs have been approved for the treatment of BCG-unresponsive NMIBC, as an adjuvant therapy, and for advanced BCa. Given the current treatment landscape, multiple clinical trials are underway to identify the optimal combinations and sequencing of agents, with the goal of further improving patient outcomes.^22–25^ Despite BCa having a high mutation burden, ICIs, as single agents, have a response rate of only around 20 %. Additionally, no reliable biomarker currently exists to identify potential responders. Multiple resistance mechanisms significantly limit the efficacy of ICIs.^26–28^

Before prescribing an ICI, a test to determine PD-L1 expression in tumor cells and tumor-infiltrating immune cells is sometimes performed.^29^ At present, immunohistochemistry (IHC) is the most commonly used method for testing PD-L1 expression.^30^ Patients who are ineligible for platinum-based chemotherapy but eligible for other chemotherapy treatments must have tumor expression of PD-L1 to receive ICI therapy as a single-agent first-line therapy.^31,32^ Yet many patients with PD-L1-expressing do not respond to ICI therapy, while some patients PD-L1-negative may still respond to immunotherapy.^26^ Thus, more reliable predictive biomarkers for ICIs are urgently needed. Since IHC staining requires cancer tissue, which is not always available, there is a growing emphasis on identifying biomarkers in non-invasive samples. This aligns with the expectation of replacing invasive cystoscopy with a non-invasive method.^33^

## 3. Urine PD-L1 as a potential biomarker

Currently, the urine PD-L1 (uPD-L1) is being explored as a non-invasive diagnostic and prognostic biomarker for BCa. To date, two methods are being developed to determine uPD-L1 of BCa. The first method is ELISA-based assay to measure the soluble-PD-L1 (sPD-L1), while the second method uses flow cytometry to assess membrane PD-L1 (mPD-L1) on cancer cells shed into urine, similar to the IHC test on tumor tissue.

### 3.1. ELISA-based urinary soluble PD-L1 (us-PD-L1) analysis

Soluble PD-L1 (sPD-L1) can originate from diverse sources. The common form is believed to arise from the cleavage of the cell membrane-bound PD-L1 (mPD-L1). Both tumor cells and activated, matured dendritic cells can release sPD-L1 through this cleavage process.^34–36^ In addition to cleaved sPD-L1, researchers have discovered that tumor cells may produce secreted PD-L1 through the alternative splice process.^37^ Both forms of sPD-L1 have the ability to bind to cell surface PD-1 and regulate tumor-induced immunosuppression.^37,38^ Thus, urine sPD-L1 (us-PD-L1) has the potential to serve as a urine biomarker for BCa and ICI therapy.^39^

Tosev et al.^40^ used the whole urine samples from BCa patients, while Vikerfos et al.^41^ studied centrifuged urine samples. Both studies reported detectable levels of us-PD-L1, with a 34.4 % detection rate for the centrifuged urine sample. However, no information was provided regarding the detection rate for whole urine samples. Both studies found that us-PD-L1 levels were significantly higher in patients diagnosed with BCa compared to the patients with non-malignant urological diseases (P < 0.05). Additionally, Vikerfos et al. also observed that us-PD-L1 levels were higher in MIBC patients than in NMIBC patients (P = 0.05). However, no significant statistical correlation was found between usPD-L1 levels and factors, such as BCa grade, stage, risk factors, or all-cause mortality.

Furthermore, Tosev et al. investigated the ability of us-PD-L1 to diagnose BCa using receiver operating characteristic (ROC) curve analysis. For newly diagnosed BCa patients, when setting the threshold for us-PD-L1 at 10.05 pg/ml, this model showed an area under the curve (AUC) of 0.78, with sensitivity and specificity values of 0.65 and 0.95, respectively. These results are similar to other potential BCa biomarkers. However, due to its limited sensitivity, us-PD-L1 is unlikely to serve as an independent diagnosis biomarker.^39^

In another study published by Ma et al., in 2023,^42^ usPD-L1 was detected in 53.45 % of whole urine samples from patients with BCa using ELISA. This us-PD-L1 level was higher in the MIBC group compared to the NMIBC group, although this difference was not statistically significant (P = 0.08). Additionally, no correlation was found between us-PD-L1 levels and the American Urological Association (AUA) defined risk stratification of NMIBC BCa, including stage, grade, or other risk factors such as smoking status.

### 3.2. Flow cytometry-based analysis of urinary cell membrane PD-L1 (mPD-L1)

As PD-L1 is a transmembrane protein expressed on the surface of both cancer cells and immune cells, flow cytometry has been used as a fast, inexpensive, and straightforward approach to detect its expression. In 2018.^43^ Chevalier et al. analyzed PD-L1 expression on Treg cells in the blood and urine samples from NMIBC patients. They found that while PD-L1 expression was barely detectable in blood samples, it was significantly enriched on Treg cells after BCG treatment. In vitro, BCG treatment induced significant increase in PD-L1+ Treg cells, and this effect was markedly amplified when co-cultured with BCa tumor cells. The induction of PD-L1 expression was mediated through the IFN-β pathway. Chevalier et al. also developed a “urine immunosuppressive score” (IS) to evaluate the role of urine Treg cells (including both conventional and novel PD-L1+ Treg cells) in predicting patients’ post-BCG recurrence. Their findings showed that a high IS correlated with significantly shorter recurrence-free survival (RFS) time.

In 2020, Alanee et al. conducted single-cell flow cytometry on urine samples, and found that the percentage of PD-L1+ white blood cells (WBC) in NMIBC patients was significantly higher than that in healthy donors (P < 0.0001). However, PD-L1 expression in epithelial cells did not differ statistically between these two patient groups.^44^ Using PD-L1 expression data and adaptive genetic algorithms, the researchers proposed a prediction model to distinguish malignancy from normal cells, achieving an area under the curve (AUC) of 90 %, with sensitivity of 98 % and specificity of 87 %.

Cantó et al.^45^ analyzed the cell population present in urine and blood samples from 24 MIBC patients at the time of radical cystectomy, as well as from 4 healthy donors. They further analyzed how the neoadjuvant chemotherapy (NAC), smoking status, and lymph nodes (LN) involvement affected immune cell composition. The percentage of PD-L1+ neutrophils in urine was positively correlated with that in matched blood samples, although the correlation was weak (r = 0.498, p = 0.015), and may not be clinically meaningful. Urinary PD-L1 + CD8^+^ cells were significantly higher in smokers compared to non-smokers (P = 0.035). Regarding LN involvement, no correlation was found between LN status and urine PD-L1+ lymphocytes. While the urine cellular composition was found to correlate with cancer recurrence, the clinical significance of these findings remains uncertain due to the small sample size.

In addition to research focused on immune cells, Wang et al.^46^ reported BCa urinary tumor cells study using flow cytometry in 2023. To test the reliability of flow cytometry as a method to detecting PD-L1 positive cells, Wang et al. compared flow cytometry results and western blot findings. The positive correlation between two methods confirmed that flow cytometry is not hindered by glycosylated PD-L1 subsets, making it a reliable and a powerful complementary tool to IHC method, which can show varying results depending on the antibody used. Innovatively, Wang et al. developed a novel prognosis model for BCa patients using a combination of conditional reprogramming (CR) technology and IFN-γ stimulation. By culturing cells through CR, they were able to obtain sufficient cells and observed that the basal expression of PD-L1 varied across different cells. Since IFN-γ plays an important role in cell immunity and can increase PD-L1 expression, they incorporated IFN-γ-stimulated PD-L1 (IFN-γ-st PD-L1) as an index to predict patients outcomes and their response to ICIs. In contrast to current The Cancer Genome Atlas (TCGA) mRNA data, which shows no prognosis effect of PD-L1 on BCa, Wang et al. found that IFN-γ-st PD-L1 levels could categorize patients to good or poor prognostic groups. Additionally, their findings suggest that urine-derived conditionally reprogrammed cells (CRCs) may provide a better prognosis function than tumor-derived CRCs. Regarding patients’ response to ICIs, Wang et al. reported the levels of IFN-γ-st PD-L1 in 8 patients after ICI treatment. Their results indicated that super high levels of IFN-γ-st PD-L1 were associated with severe immune-related adverse events (irAEs) after ICIs treatment, while moderate levels were associated with a good response (partial response, PR), and extremely low levels were associated with no response (progressive disease, PD).

In summary, through flow cytometry, we are now able to study the cell composition of BCa urine samples. By focusing on the PD-L1 positive immune cells and combining them with other cell markers, we have already identified several cell subpopulations related to BCa diagnosis, treatment response, and other risk factors. Based on these newly identified subpopulations, new models have been developed to assist in the diagnosis and prognosis of BCa. However, each new model needs to be further validated with larger patient samples. Regarding PD-L1 positive tumor cells, the study conducted by Wang et al. confirmed that CRCs can be a useful method. Compared to the baseline PD-L1 levels IFN-γ-stimulated PD-L1 (IFN-γ-st PD-L1) provided more promising data for predicting BCa prognosis and patients’ response to ICIs. This suggests that the CRCs and IFN-γ stimulation can be highly beneficial in future studies. Furthermore, Wang et al. performed RNA-Seq to analyze the related pathways, providing a solid foundation for more detailed research. However, it is important to note that while results from Wang et al. appear promising, further studies with large cohorts warrant to validate the clinical potential of IFN-γ-st PD-L1.

### 3.3. Other methods-based urine PD-L1 of BCa

In addition to flow cytometry- and ELISA-based studies of uPD-L1, two publications independently reported BCa uPD-L1 using mRNA testing of urine sediment and immunocytochemistry (ICC) of urinary tumor cells. In 2019, Miyake et al.^47^ conducted a time-course study during BCG treatment in NMIBC patients. By analyzing mRNA levels in urine sediment, they identified four genes - including PD-L1 – that were upregulated during BCG treatment. Although upregulation of PD-L1 was not statistically significant, it was associated with BCG resistance and poor prognosis. Miyake et al. further classified patients based on the number of upregulated genes and found that their model could predict the patients’ recurrence risk. Similarly, in 2023, Chen et al.^48^ reported one statistical analysis on the BCa urinary tumor cells using an immunocytochemistry based approach. By comparing PD-L1 positive tumor cells in matched tumor tissue and urine samples, they demonstrated that urinary PD-L1 expression could serve as a predictive marker for response to ICIs. Using the current clinical threshold of 25 % PD-L1-positive tumor cells in tissue as a reference, the study identified a 10 % cutoff in urine samples that yielded similar predictive value, with a sensitivity of 62.5 %, specificity of 75 %, and an AUC of 0.688.

## 4. Future potential research aspects

Cancer is a complex disease that cannot be effectively managed by a single approach; thus, identifying the optimal combination of strategies tailored to different clinical scenarios is the ultimate goal. In this context, beyond summarizing the current status of uPD-L1 research in BCa (Table 1), we also provide an overview of promising future directions for advancing uPD-L1 research.

### 4.1. Combination of conditionally reprogrammed cells (CRCs) and uPD-L1

**Fig. 1.**
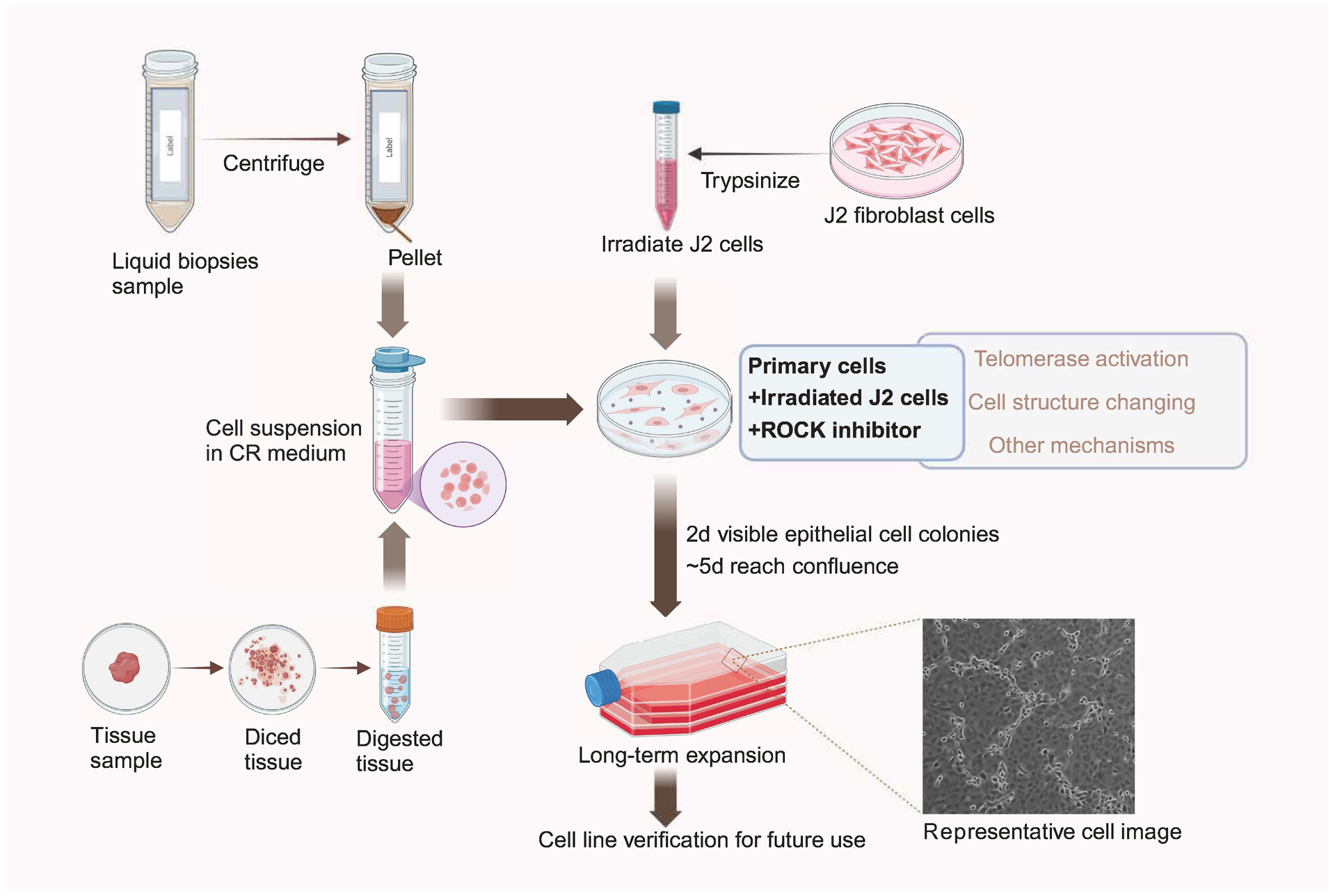
Overview of the CRC Technology for Specimen Collection and Culture System Establishment. Tissue or liquid biopsy samples are collected and processed to generate a single-cell suspension within 12 h of collection, typically maintained at 4 °C to preserve cell viability. Conditional reprogramming cells (CRCs) are then cultured through co-culture with irradiated J2 feeder cells in the presence of the ROCK inhibitor Y-27632. Epithelial cell colonies typically become visible within 2 days, and cultures usually reach confluence in approximately 5 days. As shown in the representative image, the proliferating epithelial cells are surrounded by shrunken J2 feeder cells. Long-term expansion of these cells is achievable under reversible CR culturing conditions. To ensure the authenticity of the established cell lines, validation should be performed using short tandem repeat (STR) profiling, comparative genomic hybridization (CGH), and spectral karyotyping (SKY).

**Fig. 2.**
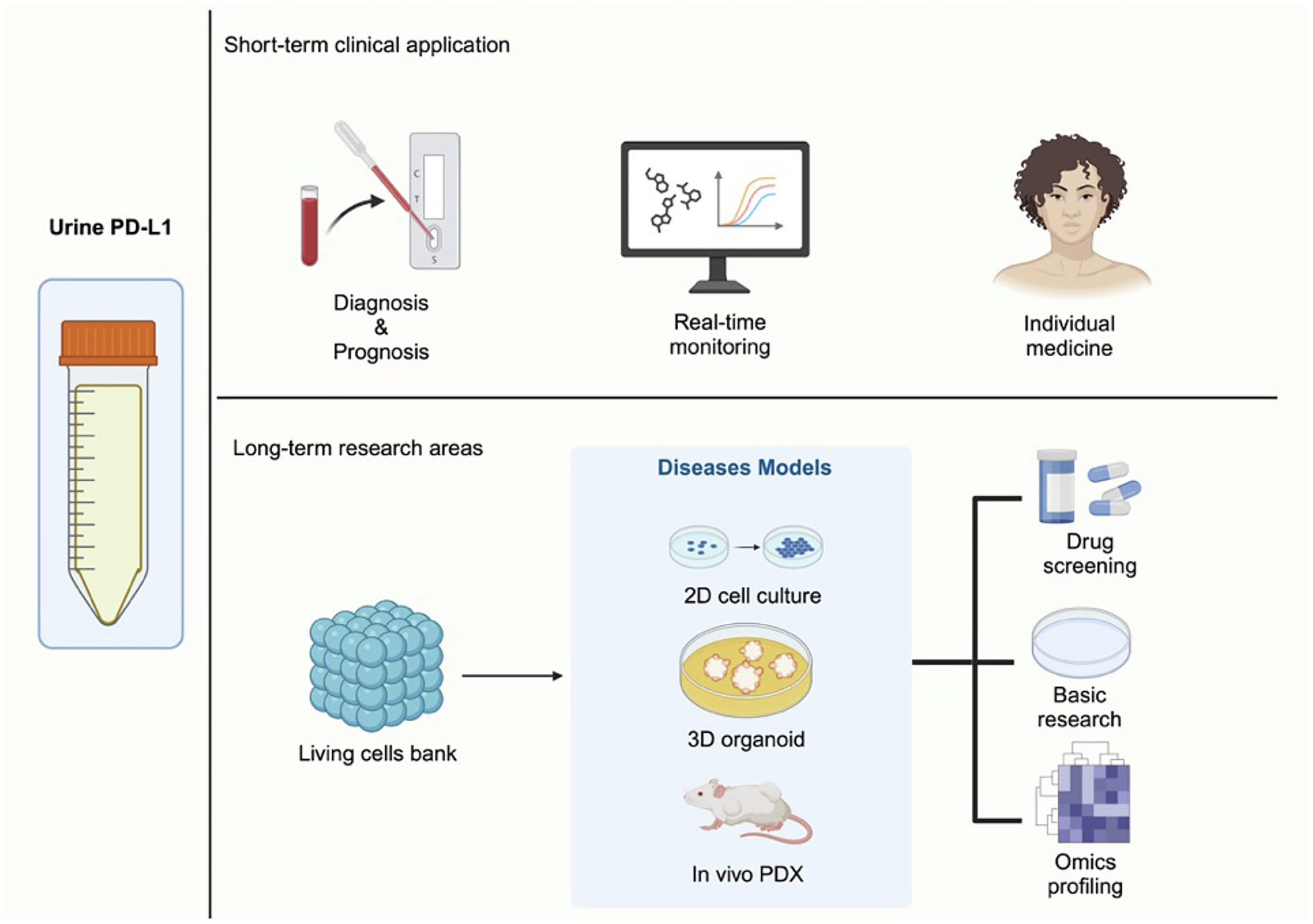
Potential Clinical Applications of Combining Conditional Reprogramming (CR) Technology with Urinary PD-L1 (uPD-L1) Analysis. Owing to its accessibility and potential accuracy, uPD-L1 holds promise as a non-invasive biomarker for early diagnosis and prognosis of bladder cancer. During the course of therapy, uPD-L1 could also serve as a dynamic indicator of patient status, enabling real-time monitoring and facilitating personalized treatment adjustments. When integrated with CR technology, large numbers of patient-derived cells can be expanded to create versatile cell banks. These cells can be used to establish a variety of *in vitro* (2D or 3D) and *in vivo* models, thereby supporting basic research, high-throughput drug screening, and comprehensive omics analyses. This combined approach has the potential to significantly enhance precision medicine in bladder cancer care.

As a next-generation cell culture method, conditionally reprogrammed cells (CRCs) have gained significant attention and have been applied in numerous basic and translational research.^49–59^ By using irradiated Swiss 3T3-J2 mouse fibroblast feeder cells in combination with ROCK inhibitor Y-27632, we can now effectively generate patient-derived normal and cancer epithelial cell lines from a variety of tissue types. Compared to other methods of cell immortalization, CRCs offer several advantages, including efficiency, preservation of the original cellular lineage, and reversibility.^60–62^ As illustrated in Fig. 1, mature CR technology enables the expansion of primary cells from either tissue or liquid biopsy samples for diverse applications. Through optimized co-culture with irradiated J2 cells and ROCK inhibitor, the amplified CRCs retain phenotypic and genotypic features consistent with the original cells. As previously discussed in reference to Wang et al., CRCs can also be effectively used in studying uPD-L1 expression. Given its promise, the potential future applications of CRCs in uPD-L1 research are discussed in the following section (Fig. 2).

Due to their rapid, efficient, and accurate characteristics, CRCs derived from patients’ urine samples represent a powerful platform for diagnosis, prognosis, and prediction of treatment response under specific therapeutic pressures. Significant progress has been made by combining CRC technology with IFN-γ stimulation to investigate uPD-L1 expression and its correlation with patient outcomes, particularly in predicting responses to ICIs. This approach represents a notable advancement over previous analyses, such as those from TCGA, which found no correlation between PD-L1 expression and bladder cancer progression. Building on this foundation, future research exploring how PD-L1 expression changes under various specific stimuli, in combination with other investigative strategies, could fill critical gaps in clinically relevant research. Ultimately, such studies may facilitate the clinical adoption of uPD-L1 as a dynamic biomarker, enabling individualized treatment planning, real-time monitoring of disease status, and timely therapeutic adjustments.

Beyond clinical applications, the combination of CRCs and PD-L1 analysis holds great promise for advancing both basic and translational bladder cancer research. CRC technology has already demonstrated utility in disease modeling, drug screening, precision medicine, and regenerative medicine. Importantly, CRCs can now be efficiently derived not only from tumor tissue but also from liquid biopsy samples such as urine—even when cell counts are too low for conventional culture methods. Jiang et al. developed a robust protocol for generating urine-derived CRCs from bladder cancer patients with high success rates, irrespective of tumor grade or patient heterogeneity. These CRCs retain key phenotypic and genotypic characteristics of the parental tumor for extended periods *in vitro*, supporting their use in long-term studies. Moreover, as demonstrated by Wang et al., transcriptomic profiling of urine-derived CRCs enables deeper investigation into the regulatory networks involving uPD-L1. This capability opens new avenues for non-invasive monitoring, mechanistic studies, and the development of novel therapeutic strategies in bladder cancer.

### 4.2. Other aspects

#### 4.2.1. Different uPD-L1 source

Urine is a complex biological fluid composed of water, dissolved solutes, cellular components, cell-derived particles, and micro-organisms. Most studies investigating uPD-L1 have used whole urine samples, which contain both cellular and non-cellular elements. However, this broad approach overlooks potential distinctions between PD-L1 sources, highlighting the need for further research into the clinical significance of PD-L1 derived from specific urine sub-components. For instance, we would expect different clinical correlations from cell-free usPD-L1 compared to mPD-L1 expressed on tumor or immune cells. Advancing uPD-L1 research in BCa will require clearly distinguishing and characterizing these individual components. Recently, attention has turned toward cell-derived extracellular vesicles (EVs), including exosomes, which mediate complex intercellular communication and reflect cellular responses to the microenvironment. Studies have shown that exosomal PD-L1 plays a role in tumor-mediated immunosuppression and is associated with response to anti-PD-1 therapy in metastatic melanoma.^63^ The presence of EVs in urine samples offers an opportunity to detect this specific form of PD-L1, along with other potential variants, which may further deepen our understanding of its biological significance and clinical utility in BCa.

#### 4.2.2. Mathematic strategies in uPD-L1 research

Bioinformatics is a potentially powerful tool that can be leveraged in this arena. Currently, proteomic and metabolomic analyses are commonly performed on soluble components of urine, while membranous components—such as cells or cell-derived particles—are preferred for transcriptomic analysis.^64^ Building on the foundation laid by Wang et al. in transcriptomic profiling of urine-derived CRCs, future studies utilizing bioinformatics have significant potential to advance the field. Furthermore, while conventional cohort studies typically require large sample sizes to ensure statistical reliability, case-control studies can serve as a valuable supplement due to their efficiency in comparing cases and controls within smaller patient groups.^65^

## 5. Conclusion

Bladder cancer is considered one of the most expensive malignancies in terms of clinical management costs.^66,67^ There is an urgent need for cost-effective methods for diagnosis and prognosis in this field. Urine, a non-invasive and easily obtainable biospecimen, holds great potential for biomarker analysis in bladder cancer management. Among the promising biomarkers under investigation, uPD-L1 stands out as a potential milestone in bladder cancer therapy. Ongoing research is actively exploring the clinical utility of uPD-L1, which may serve as a foundation for advancing its role in the diagnosis, prognosis, and prediction of response to ICIs in patients with bladder cancer.

## Figures and Tables

**Table 1:** List of the studies in the literature on urine PD-1/PD-L1 in BCa.

	Description	Specify diseases status	Significance & Model quality	Reference
Diagnosis	usPD-L1, BCa > non malignancy patients	BCa	P < 0.05	^40,41^
	usPD-L1, MIBC > NMIBC	BCa	P ≥ 0.05	^41,42^
	PD-L1+ WBC, NMIBC > healthy donors	NMIBC	P < 0.0001	^44^
Prognosis	IFN-γ-st PD-L1, good outcome > bad outcome	BCa	P < 0.05	^46^
	PD-L1+ neutrophils, recurrent > non recurrent	LN-, non-NAC treated MIBC	P = 0.021	^45^
	PD-L1 mRNA, bad outcome > good outcome	post-BCG BCa	none	^47^
ICIs response prediction	IFN-γ-st PD-L1, the level increases as patients irAEs degree	ICIs treated BCa	–	^46^
	PD-L1+ tumor cells, 10 % as a threshold to predict ICIs response	BCa	Sensitivity 62.5 % Specificity 75 % AUC 0.688	^48^
Correlation with treatment & risk factor	PD-L1+ Treg, post-BCG > pre-BCG	NMIBC	P ≤ 0.01	^43^
	PD-L1+ neutrophils, NAC treated > non-NAC treated	MIBC	P = 0.041	^45^
	matched blood result		r = 0.498, p = 0.015	
	PD-L1+ CD8^+^ cells, smoker > non-smoker	MIBC	P = 0.035	^45^
	usPD-L1, age	BCa	P < 0.0001	^40^
	usPD-L1, IC-treated > non-IC treated	BCa	P < 0.05	^42^
	usPD-L1, non-CIS > CIS	BCa	P < 0.05	^42^
	usPD-L1, AD > non-AD	BCa	P < 0.05	^42^
Involved multi-factor model	Urine immunosuppressive score, short RFS > long RFS	post-BCG recurrence	P = 0.007	^43^
	adaptive genetic algorithms, distinguish malignant phenotype from normal cells	NMIBC	Sensitivity 98 % Specificity 87 % AUC 0.9	^44^
	multifactorial Cox regression analysis	MIBC recurrence	P < 0.001 HR 3.51	^42^

Abbreviations: IC: intravenous chemotherapy; CIS: carcinoma in situ; AD: aberrant differentiation.
